# Industrial Wireless Sensor Networks: Protocols and Applications

**DOI:** 10.3390/s20205809

**Published:** 2020-10-14

**Authors:** Seong-eun Yoo, Taehong Kim

**Affiliations:** 1 School of Computer and Communication Engineering, Daegu University, Gyeongsan 38453, Korea; seyoo@daegu.ac.kr; 2 School of Information and Communication Engineering, Chungbuk National University, Cheongju 28644, Korea

**Keywords:** protocols for Industrial Wireless Sensor Networks (IWSN), IWSN standards, IEEE 802.15.4, WirelessHART, ISA100.11a, real-time communications in IWSN, Wireless Networked Control Systems (WNCSs), IWSN testbeds and applications

## Abstract

Wireless sensor networks are penetrating our daily lives, and they are starting to be deployed even in an industrial environment. The research on such industrial wireless sensor networks (IWSNs) considers more stringent requirements of robustness, reliability, and timeliness in each network layer. This Special Issue presents the recent research result on industrial wireless sensor networks. Each paper in the special issue has unique contributions in the advancements of industrial wireless sensor network research and we expect each paper to promote the relevant research and the deployment of IWSNs.

## 1. Introduction

With the help of technological advances and standardization activities, we are witnessing the growth of wireless sensor network applications in many areas, such as home appliances, agriculture, transportation, and manufacturing. Recently, the adoption of wireless sensor networks (WSNs) in industrial areas has increased due to matured technologies such as transceivers and protocols. Industrial wireless sensor networks (IWSNs) require reliability, robustness, and timeliness in the information exchange between devices, and they need more sophisticated protocols that many researchers and developers are working on.

This Special Issue focuses on the latest research, application, and adoption of wireless sensor networks in industrial fields from the perspective of protocols and applications, requiring high reliability and real-time packet delivery. In such a context, this Special Issue invited contributions in the following topics (though without being limited to them):

- Protocols for industrial wireless sensor networks
- Wireless network control systems
- Real-time communications in IWSNs
- IWSN testbeds and applications such as smart factories
- Edge and fog computing for IWSN
- Any subjects relevant to IWSN.

There were number of submissions and two to four reviewers evaluated each submitted article and finally six outstanding papers were accepted and published in this Special Issue. We present the brief summary of each accepted paper in the following section.

## 2. Brief Review of the Published Articles

The first paper [1] on the network layer presents an adaptive real-time routing protocol for an (*m*, *k*)-firm real-time model. The model defines the real-time requirement that at least *m* out of any *k* consecutive messages from a real-time stream must meet their deadlines to ensure an adequate Quality of Service (QoS). The paper proposes a new adaptive path selection and traffic shaping algorithm. The evaluation in OPNET shows that the proposed scheme can reduce the dynamic failure regardless of the background traffic while reducing the energy consumption.

The next two papers are relevant to Media Access Control (MAC) sub-layer. To provide a remedy for the joint problem of the low-access efficiency and the interference diffusion in high-density deployment of IWSNs, the second paper [2] on MAC (Media Access Control) proposes a spatial group-based multi-user Full Duplex OFDMA (GFDO) protocol. While the theoretical analysis derives the average number of nodes in an access channel, system saturation throughput, and area throughput, the simulation in an NS2 simulator supports the theoretical analysis and shows the efficiency of the proposed protocol.

Another paper [3] related to the MAC sub-layer issue proposes a novel distributed scheduling scheme for an ad-hoc network. The proposed scheme consists of two steps of reallocation procedures: intra-node and inter-node. The intra-node slot scheduling reallocates the packets in priority-based multiple queues using a self-fairness index to increase the throughput and delay, while the inter-node scheduling reallocates slots between neighboring nodes to increase fairness. The simulation study with a Java-based network simulator shows that the proposed scheme can adjust the packet delivery performance according to a predefined weight factor and outperform the conventional algorithms in throughput and delay.

Time synchronization is another important research area in IWSNs. The paper on the time synchronization issue proposes an energy-efficient reference node selection (EERS) algorithm [4]. EERS minimizes the number of connected reference nodes and keeps a minimal number of hops. The evaluation in an experimental network with 25 real hardware platforms shows EERS needs fewer messages (i.e., less energy consumption) than the existing techniques such as R-Sync [5], FADS [6], and LPSS [7]. In addition, the simulation study with MATLAB for a larger scale network evaluation shows that the proposed protocol provides energy efficiency without losing the accuracy of the time synchronization.

There are two papers related to the popular IWSN standards: IEEE 802.15.4 and ISA 100.11a. The first paper [8] considers the event monitoring in hazards and disaster detection in a large area with the IEEE 802.15.4/ZigBee standard. This paper proposes a dynamic reconfiguration mechanism of cluster-tree IWSNs. The proposed algorithm can dynamically reconfigure large-scale IEEE 802.15.4 cluster-tree IWSNs to assign communication resources to the overloaded branches of the tree based on the accumulated network load generated by each of the sensor nodes. The simulation assessment with CT-Sim [9] shows that the proposed scheme can guarantee the required quality of service level for the dynamic reconfiguration of cluster-tree networks.

The last paper [10] presents a wireless network control system (WNCS) composed of ISA 100.11a-based field devices (i.e., sensor nodes), a network manager, a controller, and a wired actuator. The system controls the liquid level in the tank of the coupled tank system. In order to assess the influence of the sensor link failure on the control loop, the controller calculates the link stability and chooses an alternative link in case of instability in the current link. Preliminary experimental tests of WNCS performance shows that the link stability factor allows a prediction of the change in the control system error. Finally, the tests of the control system based on link stability show that the link stability metric is able to identify possible instabilities and prevent the failure of the system’s control loop. Even with disturbances in the network links, the control system error remains below the threshold.
